# Cinobufagin induces acute promyelocytic leukaemia cell apoptosis and PML-RARA degradation in a caspase-dependent manner by inhibiting the β-catenin signalling pathway

**DOI:** 10.1080/13880209.2022.2118792

**Published:** 2022-09-19

**Authors:** Yaoyao Bian, Mei Xue, Xinlong Guo, Wenjuan Jiang, Ye Zhao, Zhaofeng Zhang, Xian Wang, Yongkang Hu, Qi Zhang, Wenliang Dun, Liang Zhang

**Affiliations:** a Jiangsu Key Laboratory for Pharmacology and Safety Evaluation of Chinese Materia Medica, School of Pharmacy, Nanjing University of Chinese Medicine, Nanjing, China; b College of Basic Medical Sciences, Institute of TCM-related Comorbid Depression, Nanjing University of Chinese Medicine, Nanjing, China; c Department of Pharmacy, Nanjing Hospital of Chinese Medicine Affiliated to Nanjing University of Chinese Medicine, Nanjing, China

**Keywords:** Haematopoietic malignancy, promyelocytic leukaemia–retinoic acid receptor A, traditional Chinese medicine

## Abstract

**Context:**

Acute promyelocytic leukaemia (APL) is a malignant hematological tumour characterized by the presence of promyelocytic leukaemia–retinoic acid receptor A (PML-RARA) fusion protein. Cinobufagin (CBG) is one of the main effective components of toad venom with antitumor properties. However, only a few reports regarding the CBG treatment of APL are available.

**Objective:**

We explored the effect and mechanism of action of CBG on NB4 and NB4-R1 cells.

**Materials and methods:**

We evaluated the viability of NB4 and NB4-R1 cells treated with 0, 20, 40, and 60 nM CBG for 12, 24, and 48 h. After treatment with CBG for 24 h, Bcl-2 associated X (Bax), B-cell lymphoma 2 (Bcl-2), β-catenin, cyclin D1, and c-myc expression was detected using western blotting and real-time polymerase chain reaction. Caspase-3 and PML-RARA expression levels were detected using western blotting.

**Results:**

CBG inhibited the viability of NB4 and NB4-R1 cells. The IC_50_ values of NB4 and NB4-R1 cells treated with CBG for 24 h were 45.2 nM and 37.9 nM, respectively. CBG induced NB4 and NB4-R1 cell apoptosis and PML-RARA degradation in a caspase-dependent manner and inhibited the β-catenin signalling pathway.

**Discussion and conclusion:**

CBG induced NB4 and NB4-R1 cell apoptosis and PML-RARA degradation in a caspase-dependent manner by inhibiting the β-catenin signalling pathway. This study proposes a novel treatment strategy for patients with APL, particularly those with ATRA-resistant APL.

## Introduction

Acute promyelocytic leukaemia (APL), also known as acute myeloid leukaemia type M3, is a haematopoietic malignancy characterized by the accumulation of large amounts of immature blood cells in haematopoietic tissues such as bone marrow. APL pathogenesis involves the translocation of chromosomes 15 and 17, which generates the promyelocytic leukaemia–retinoic acid receptor A (PML-RARA) fusion protein (Yilmaz et al. 2021). PML-RARA has dual functions of interfering with transcription regulation and destroying promyelocytic leukaemia homeostasis (Noguera et al. 2019). It can prevent normal differentiation of promyelocytic cells, block cell apoptosis, and stop at the promyelocytic stage, leading to APL occurrence. Moreover, PML-RARA has also become a significant diagnostic marker for APL (Wang et al. 2016). The degradation of PML-RARA fusion protein, which occurs via the autophagy–lysosomal (Isakson et al. 2010; Zeng et al. 2014), ubiquitin–proteasome (Rabellino et al. 2012), and caspase-dependent pathways (Wang et al. 2016), induces the differentiation of APL cells, thereby alleviating the disease (Kogan and Bishop 1999). Early, untreated APL is a dangerous, rapidly progressing, and lethal condition. Arsenic trioxide (ATO) and all-*trans-*retinoic acid (ATRA) significantly improve the prognosis of patients with APL. However, ATO and ATRA may cause adverse reactions, such as leukocytosis, infection, and heart, liver, and renal toxicity, and some patients develop ATRA resistance (Yamakawa et al. 2021). ATRA resistance affects the prognosis of patients, which is a serious obstacle in current clinical treatment. Therefore, highly efficient natural drugs with low toxicity that can overcome ATRA resistance are urgently needed to treat patients with APL.

In recent years, the treatment of cancer with natural products has gradually attracted attention (Liu et al. 2021). Cinobufagin (CBG) is extracted from toad venom, a traditional Chinese medicine with numerous functions, such as anti-inflammatory and antiviral functions, and is used as a cardiac glycoside. Recent studies have found that CBG plays an antitumor role in melanoma (Pan et al. 2019; Kim et al. 2020; Zhang et al. 2020), colorectal (Li et al. 2019) and nasopharyngeal (Pan et al. 2020) cancers, osteosarcoma (Ma et al. 2016), and other malignant tumours by inducing apoptosis (Pan et al. 2020; Zhang et al. 2020), blocking tumour angiogenesis (Li et al. 2019), and arresting cell cycle (Pan et al. 2019, 2020), and autophagy (Ma et al. 2016). However, only a few studies have reported the effects of CBG treatment on APL.

To determine whether CBG had an anti-APL effect on ATRA-sensitive and -resistant APL cell lines, we employed the NB4 (ATRA-sensitive) and NB4-R1 (ATRA-resistant) APL cell lines. NB4-R1 is a common ATRA-resistant APL cell model which has been used in various studies (Ge et al. 2014; Liang et al. 2019). In this study, we investigated the effects of CBG on ATRA-sensitive and -resistant APL cells with the aim of providing an experimental basis for the clinical treatment of ATRA-resistant APL.

## Materials and methods

### Materials

CBG (purity ≥98%, B20542) was purchased from Yuanye Biotechnology Co., LTD. (Shanghai, China), Z-VAD-FMK (A1902) and cycloheximide (CHX, A8244) were purchased from ApexBio (Houston, TX, USA), Suc-Leu-Leu-Val-Tyr-AMC (ab142120) and PML-RARA antibody (AB43152) were purchased from Abcam (Cambridge, MA, USA), lithium chloride (LiCl, L9650) was purchased from Sigma-Aldrich (St. Louis, MO, USA), cleaved caspase-3 (9661 T) and β-catenin (8480S) antibodies were purchased from Cell Signalling Technology (Danvers, MA, USA), microtubule-associated protein light chain 3 (LC3, 4600-1-AP), p62 (18420-1-AP), Bax (50599-2-Ig), Bcl-2 (12789-1-AP), Pro-caspase 3 (19677-1-AP), cyclin D1 (60186-1-Ig), c-myc (10828-1-AP), C/EBP homologous protein (CHOP, 60304-1-Ig), eukaryotic initiation factor 2α (eIF2α, 66482-1-Ig), protein kinase R-like endoplasmic reticulum kinase (PERK, 24390-1-AP), and β-actin (20536-1-AP) antibodies were purchased from Proteintech (Chicago, IL, USA), and p-eIF2α (Ser51) (AF3087), p-PERK (Thr982) (DF7576), and activating transcription factor 4 (ATF4, DF6008) antibodies were purchased from Affinity Biosciences (Cincinnati, OH, USA).

### Cell culture

The ATRA-sensitive APL cell line (NB4 cells) was purchased from BeNa Culture Collection, and the ATRA-resistant APL cell line (NB4-R1 cells) was kindly provided by the Institute of Haematology, Ruijin Hospital, Shanghai Jiao Tong University School of Medicine. The NB4 and NB4-R1 cells were grown in Roswell Park Memorial Institute 1640 medium (Gibco, Grand Island, NY, USA), supplemented with 10% foetal bovine serum (Gibco), and grown in an incubator with 37 °C and 5% carbon dioxide.

### Cell viability assay

Cells (5 × 10^4^ cells/mL, 200 μL) were seeded into 96-well plates and treated with different CBG doses for 24 h. Thereafter, 20 μL cell counting kit-8 (ApexBio, Houston, TX, USA) was added and incubated at 37 °C for 1-4 h. The absorbance of the 96-well plate was measured at a wavelength of 450 nm using a microplate reader (Tecan, Männedorf, Switzerland).

### Lactate dehydrogenase (LDH) activity assay

NB4 and NB4-R1 cells were seeded at 1 × 10^5^ cells/mL into 6-well plates, treated with different CBG concentrations, and incubated with serum-free media for 24 h. Thereafter, the cells were collected and centrifuged (3000 rpm, 5 min) to extract the supernatant. Supernatant LDH activity was measured using the LDH assay kit (Beyotime Biotechnology, Shanghai, China).

### Hoechst 33258 staining

Hoechst 33258 staining kit (Beyotime) was used for apoptosis analysis. After 24 h of CBG treatment, cells were collected and stained with Hoechst 33258 staining solution for 5 min and placed on slides to observe under a fluorescence microscope (Nikon, Tokyo, Japan).

### Flow cytometry analysis

To detect apoptosis using Annexin V-fluorescein isothiocyanate (FITC)/propidium iodide (PI) double staining (Vazyme, Nanjing, Jiangsu, China), cells were inoculated at 10^6^ cells/mL in Petri dishes containing different drug concentrations, incubated for 24 h, and collected. Annexin V-FITC and PI staining solution were added to each sample, which was incubated in the dark. Apoptosis was detected using flow cytometry (Beckman Coulter LTD, Brea, CA, USA).

### Western blotting

Cells were inoculated at 10^6^ cells/mL in Petri dishes containing different drug concentrations, incubated for 24 h, and then collected and lysed with lysis buffer (radioimmunoprecipitation assay buffer: phenylmethylsulfonyl fluoride: protein phosphatase inhibitor = 100:1:1). The protein concentration was determined using a bicinchoninic acid (BCA) protein concentration determination kit (Beyotime). Proteins were separated using sodium dodecyl sulfate-polyacrylamide gel electrophoresis and transferred to a nitrocellulose membrane (Micon, Miller, MA, USA), which was blocked with Tris-buffered saline with Tween (TBST) containing 5% bovine serum albumin (BSA) for 1 h, washed with TBST, and incubated with primary antibody at 4 °C overnight. Thereafter, the membrane was incubated with secondary antibody in TBST for 1 h and washed, and the protein bands were visualized using a gel imager (Bio-Rad, Hercules, CA, USA).

### Immunofluorescence

NB4 and NB4-R1 cells were treated with drugs for 24 h, collected, washed with phosphate-buffered saline (PBS), and fixed with 4% paraformaldehyde. Thereafter, the cells were permeabilized with 0.1% TritonX-100, incubated with 5% BSA at 25 °C, and incubated with antibodies at 4 °C overnight. The cells were then washed, incubated with secondary antibodies, and stained with 4′,6-diamidino-2-phenylidene for fluorescence microscope (Nikon).

### JC-1 staining

Mitochondrial membrane potential (MMP) was measured using an MMP measurement kit (JC-1, Beyotime). The cells were collected after centrifugation, washed, and incubated with JC-1 staining solution at 37 °C for 30 min, followed by fluorescence microscopy (Nikon).

### Reactive oxygen species (ROS) activity detection

The cells were collected and incubated with 2′, 7′-dichlorodihydrofluorescein diacetate (Beyotime) for 30 min, and ROS activity was detected using a fluorescence microscope (Nikon) or fluorescence microplate reader (Tecan) with excitation at 488 nm and emission at 525 nm.

### 20s proteasome activity

After CBG treatment for 24 h, cells were collected, washed, and lysed with lysis buffer without protease inhibitors. Protein concentration was measured using a BCA kit (Beyotime). The same amounts of protein and substrate (Suc-Leu- Leu-Val-Tyr-AMC) were co-incubated at 37 °C for 1 h, and AMC release was detected using a fluorescence microplate reader (Tecan) with excitation at 355 nm and emission at 460 nm.

### Ca^2+^ activity assay

After 24 h of CBG treatment, cells were collected, washed, and stained with 4 μM Fluo-4 (Beyotime) staining solution at 37 °C for 30 min in the dark. Thereafter, the cells were washed with PBS and incubated for 20 min. Ca^2+^ activity was then detected using a fluorescence microscope (Nikon) or fluorescence microplate reader (Tecan) with excitation at 488 nm and emission at 515 nm.

### Real-time polymerase chain reaction (RT-PCR)

In an RNA-free environment, RNA was extracted from cells using TRIzol reagent (Invitrogen, Carlsbad, CA, USA) according to the manufacturer’s instructions. The RNA was then reverse transcribed using RT SuperMix for the qPCR reverse transcription kit (Vazyme, Nanjing, Jiangsu, China). Thereafter, cDNA was amplified using the ChamQ Universal SYBR qPCR Master Mix reagent. The gene expression of each sample was normalized to glyceraldehyde 3-phosphate dehydrogenase (GADPH) expression as an internal control, and the expression of each target gene was calculated using the 2^−ΔΔCT^ method. The primers used for amplification are as follows:

Bax
Primer F 5′-CCCGAGGTCTTTTTCCGAG-3′;
Primer R 5′-CCAGCCCATGATGGTTCTGAT-3′
Bcl-2
Primer F 5′-GGTGGGGTCATGTGTGTGG-3′;
Primer R 5′-CGGTTCAGGTACCTCAGTCATCC-3′
CHOP
Primer F 5′-CCTCACTCTCCAGATTCCAG-3′;
Primer R 5′-GCCACTTTCCTTTCATTCTC-3′
β-catenin
Primer F 5′-CCTATGCAGGGGTGGTCAAC-3′;
Primer R 5′-CGACCTGGAAAACGCCATCA-3′
c-myc
Primer F 5′- TCTTCCCCTACCCTCTCAAC-3′;
Primer R 5′-AGCCTGCCTCTTTTCCAC-3′
cyclin D1
Primer F 5′-GAGCCCGTGAAAAAGAGC-3′;
Primer R 5′-GGAGTTGTCGGTGTAGATGC-3′
GAPDH
Primer F 5′-CACCATCTTCCAGGAGCGAG-3′;
Primer R 5′-AAATGAGCCCCAGCCTTCTC-3′

### Statistical analysis

Statistical analysis was performed with GraphPad Prism 9 software (GraphPad Software Inc. CA, USA). All data are expressed as mean ± standard deviation. *t*-Test was used to compare the two groups, and one-way analysis of variance (ANOVA) was used to compare multiple groups. *P* < 0.05 was considered to indicate significance.

## Results

### CBG inhibited the viability of NB4 and NB4-R1 cells

**Figure 1.**
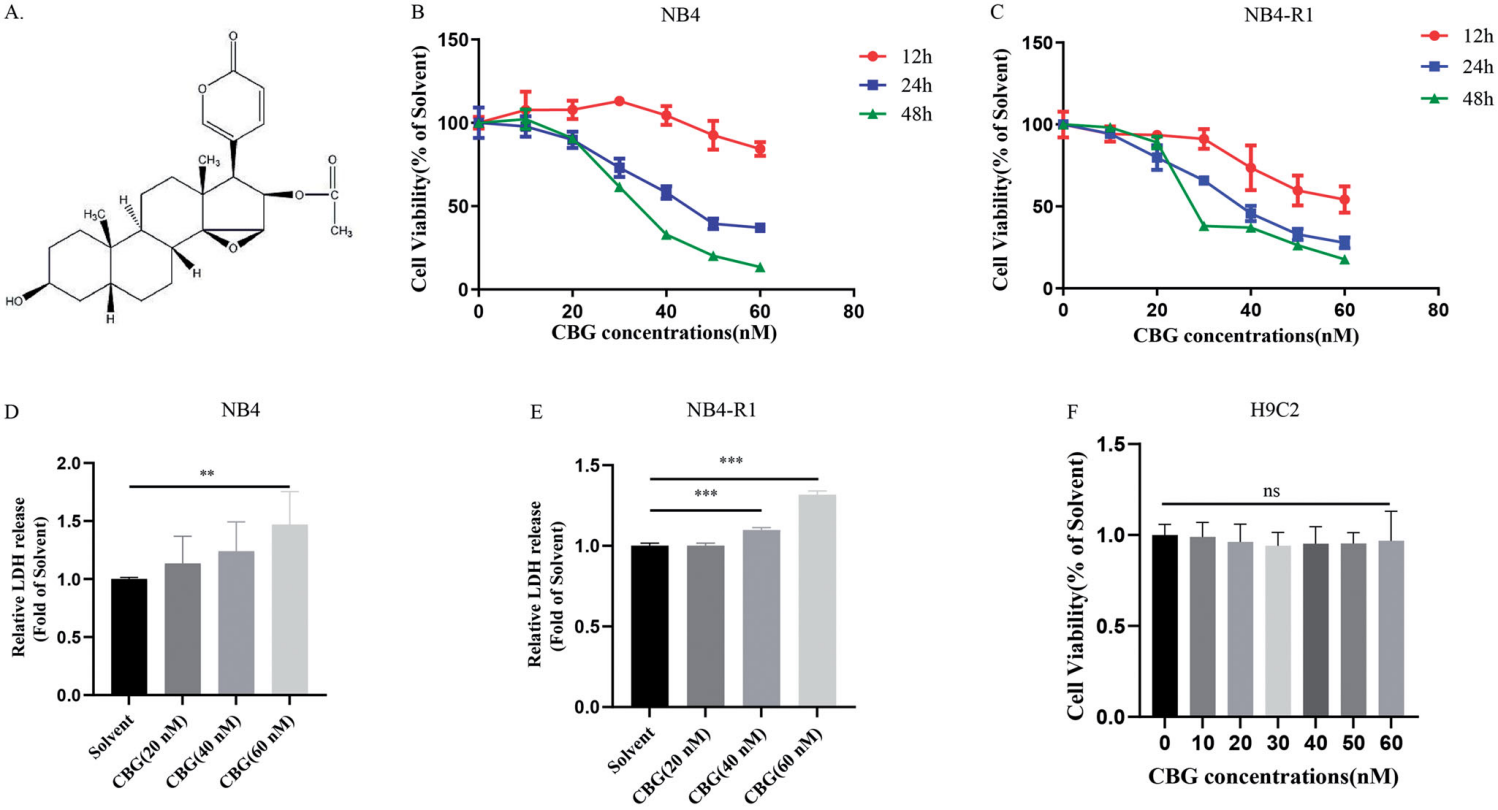
CBG inhibited the activity of NB4 and NB4-R1 cells. (A) Chemical structure of CBG. (B, C) NB4, NB4-R1 cells were treated with CBG for 12 h, 24 h, and 48 h to detect cell viability. (D, E) NB4, NB4-R1 cells were treated with CBG for 24 h to detect the LDH release. (F) The effect of CBG on the viability of H9C2 cells treated at 24 h. The data are expressed as mean ± SD (n = 6). Compared with solvent group, *P < 0.05, **P < 0.01, ***P < 0.001. ns: no significant difference.

The structural formula of CBG is shown in Figure 1(A). The viability of NB4 and NB4-R1 cells was first detected after 12, 24, and 48 h of CBG treatment. Notably, CBG inhibited the viability of NB4 and NB4-R1 cells in a time- and dose-dependent manner (Figure 1(B,C)). CBG (20, 40, and 60 nM) was co-incubated with NB4 and NB4-R1 cells for 24 h, resulting in an increase in LDH release (Figure 1(D,E)). Furthermore, CBG induces myocardial toxicity (Xiong et al. 2019). However, we found that CBG had no significant effect on the myocardial cell line (H9C2 cells) after 24 h of treatment at CBG concentrations of 0–60 nM (Figure 1(F)).

### CBG induced apoptosis and inhibited PML-RARA expression in NB4 and NB4-R1 cells

**Figure 2.**
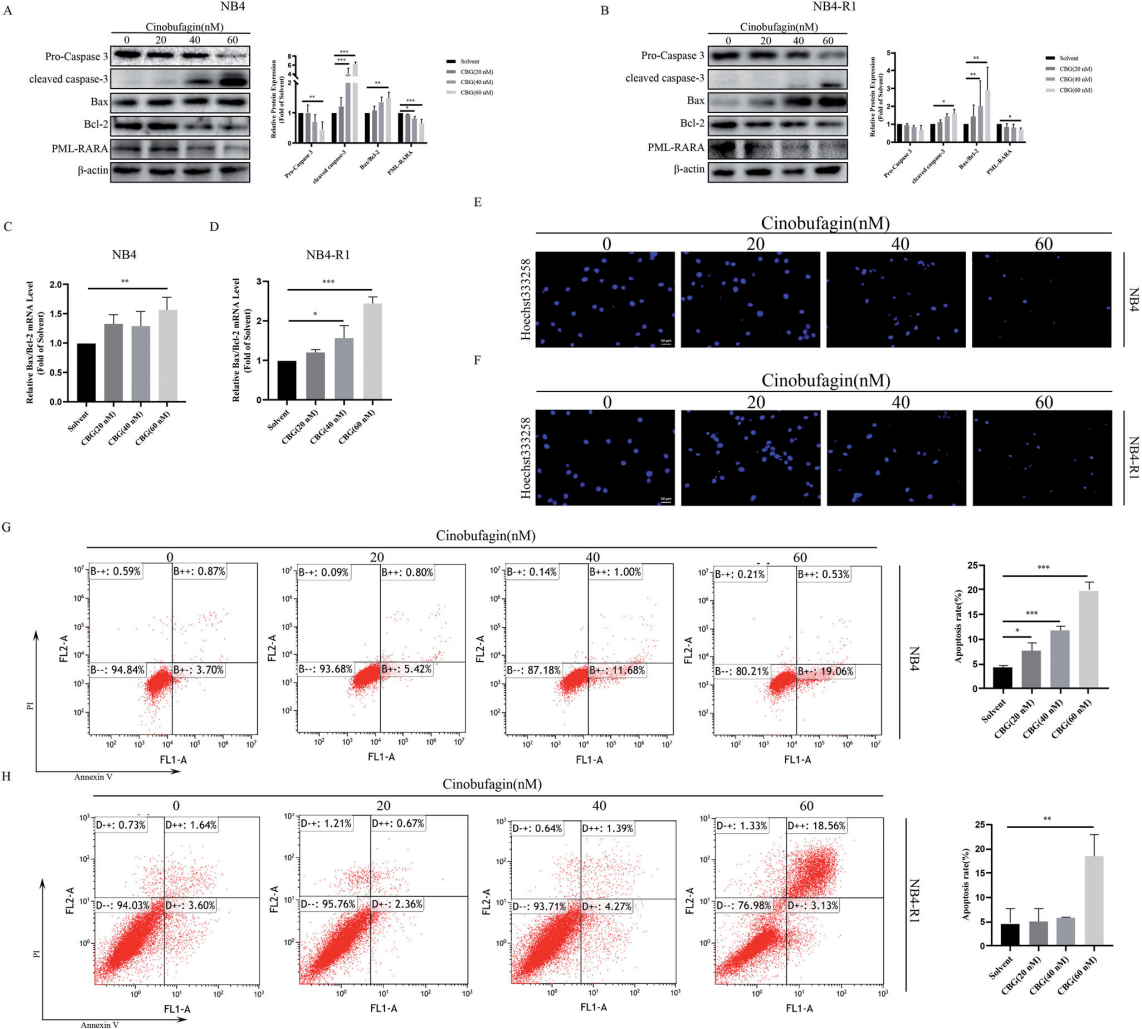
CBG induced apoptosis of NB4 and NB4-R1 cells. (A, B) NB4 and NB4-R1 cells were treated with CBG for 24 h. Apoptosis related protein expression (Pro-Caspase 3, cleaved caspase-3, Bax, Bcl-2) and PML-RARA were detected by western blotting. (C, D) RT-PCR was used to detect the expression of *Bax* and *Bcl-2* in NB4 and NB4-R1 cells after administration of CBG (0-60 nM) for 24 h. (E, F) Hoechst staining observed nucleus fragmentation of NB4 and NB4-R1 cells. (G, H) Annexin V-FITC/PI double staining was used to detect the apoptosis ratio. The results are expressed as mean ± SD (n = 3). Compared with solvent group, *P < 0.05, **P < 0.01, ***P < 0.001.

**Figure 3.**
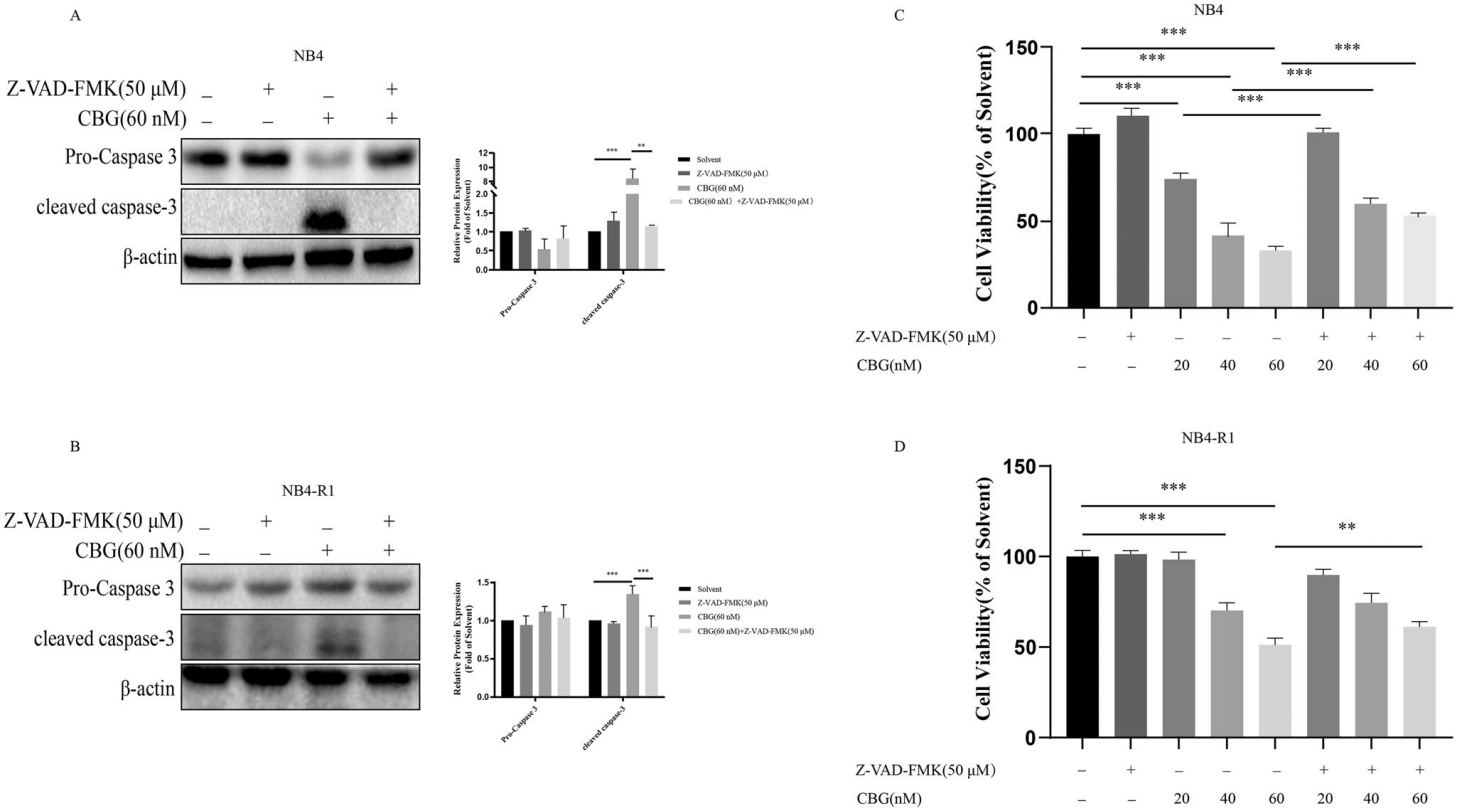
Apoptosis inhibitor Z-VAD-FMK inhibited CBG-induced cell death. NB4 and NB4-R1 cells were pre-treated with Z-VAD-FMK for 2 h and then incubated with CBG (0–60 nM) for 24 h. (A, B) Western blotting was used to detect pro-caspase 3 and cleaved caspase-3. The data are expressed as mean ± SD (n = 3). (C, D) The activity of NB4 and NB4-R1 cells was detected by CCK-8. The results are expressed as mean ± SD (n = 6). Compared with the CBG group with a specified dose, *P < 0.05, **P < 0.01, ***P < 0.001.

Cleaved caspase-3 was detected after the administration of CBG. (Figure 2(A,B)). Bax and Bcl-2 are key apoptotic proteins of the Bcl family, and the Bax/Bcl-2 ratio is an important indicator of apoptosis. Western blotting results revealed that the Bax/Bcl-2 ratio was significantly increased after CBG treatment (Figure 2(A–D)). Following CBG administration, NB4 and NB4-R1 cells exhibited nucleus fragmentation (Figure 2(E,F)) and increased apoptosis (Figure 2(G,H)). Z-VAD-FMK, a broad-spectrum caspase inhibitor, inhibited cleaved caspase-3 (Figure 3(A,B)) and increased cell viability after CBG treatment (Figure 3(C,D)). We also found that CBG treatment inhibited PML-RARA expression (Figure 2(A,B)). These data suggested that CBG promoted apoptosis in a caspase-dependent manner and inhibited PML-RARA expression in NB4 and NB4-R1 cells.

### CBG-induced apoptosis in NB4 and NB4-R1 cells was accompanied by mitochondrial damage

**Figure 4.**
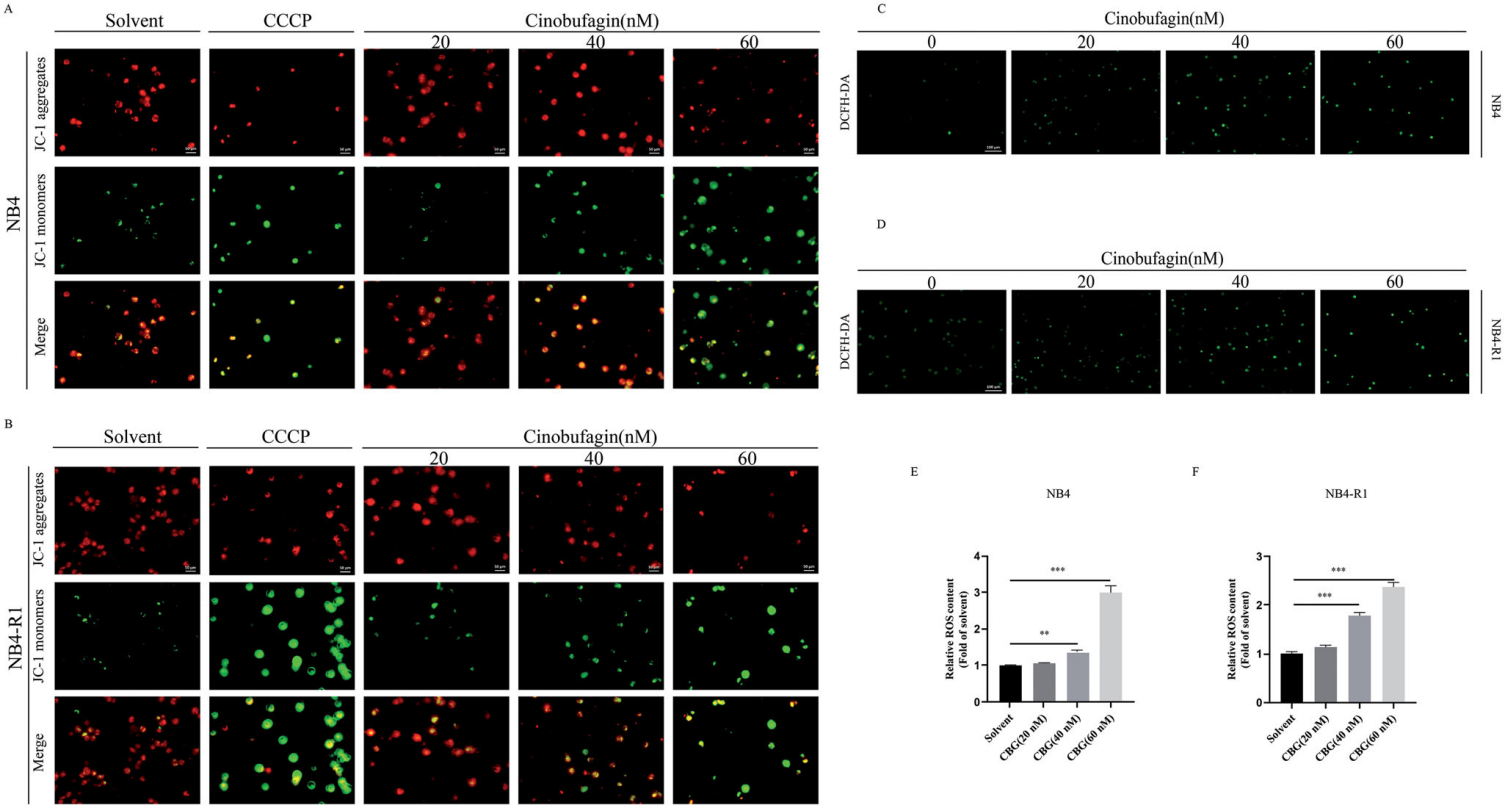
Mitochondrial damage and reactive oxygen species increased in NB4 and NB4-R1 cells induced by CBG. (A, B) NB4, NB4-R1 cells were treated with (0–60 nM) CBG for 24 h, and the changes of mitochondrial membrane potential were detected by JC-1. The red cells represented higher membrane potential, and the green cells represented lower membrane potential, with the scale of 100 μm. ROS expression was observed by (C, D) fluorescence microscope (scale 100 μm) and (E, F) fluorescence quantitative microplate. The results are expressed as mean ± SD (n = 3). Compared with solvent group, *P < 0.05, **P < 0.01, ***P < 0.001.

Apoptosis is often accompanied by mitochondrial damage, and the decrease in MMP is a sign of mitochondrial damage (Zhu et al. 2021). To further explore whether CBG caused mitochondrial damage, we measured MMP, interestingly, MMP decreased (Figure 4(A,B)). Mitochondria are the main sites of ROS production, and increased ROS might reflect mitochondrial damage. Further experiments revealed a large amount of ROS in NB4 and NB4-R1 cells after CBG administration (Figure 4(C–F)). These results suggested that CBG induced mitochondrial damage in NB4 and NB4-R1 cells.

### CBG induced endoplasmic reticulum (ER) stress in NB4 and NB4-R1 cells

**Figure 5.**
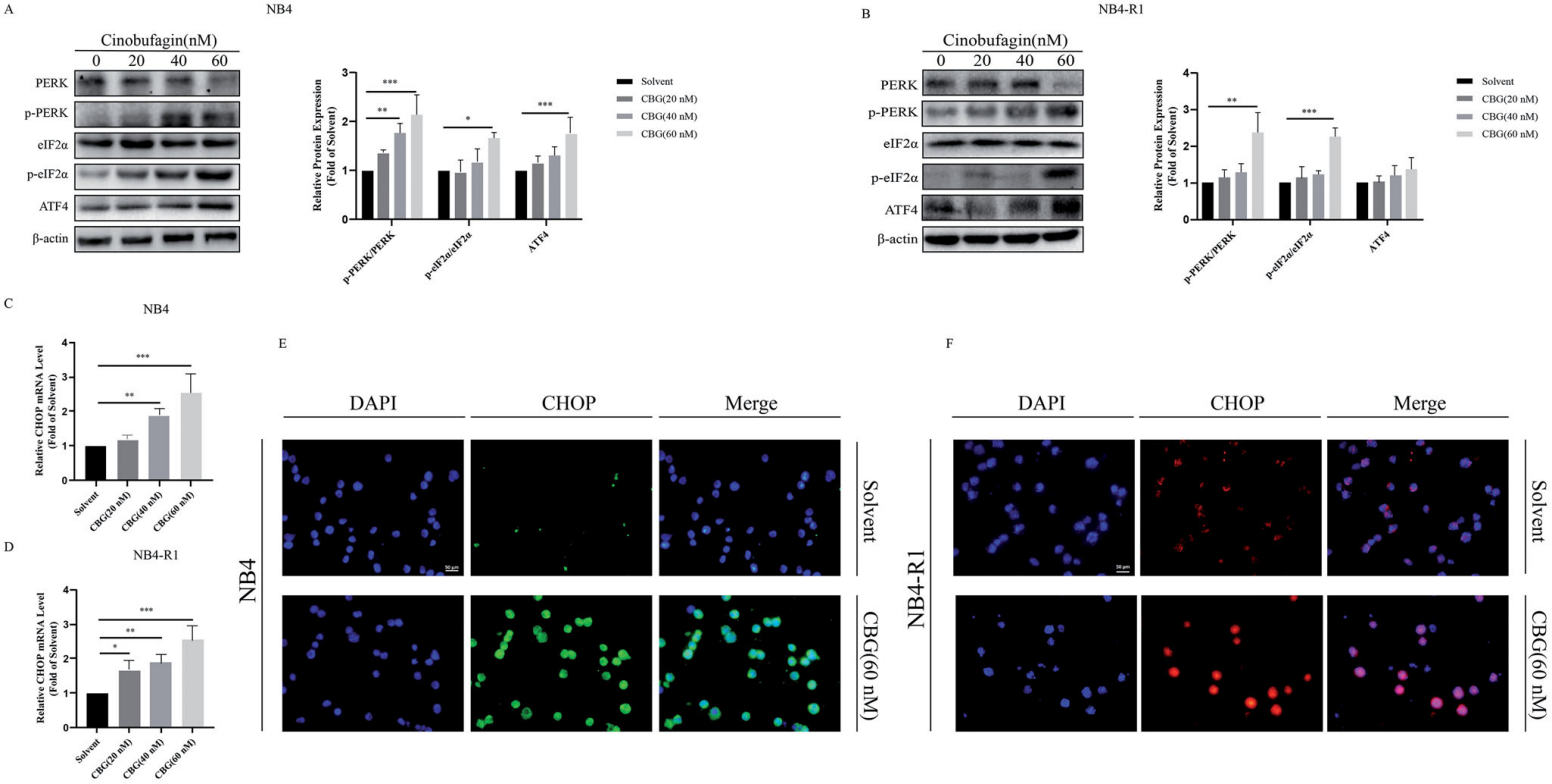
CBG stimulated ER stress. (A, B) NB4 and NB4-R1 cells were treated with different concentrations of CBG for 24 h, western blotting detects the PERK-eIF2α-ATF4 expression. (C, D) RT-PCR was used to measure the *CHOP* expression. Results are expressed as mean ± SD (n = 3). Compared with solvent group, *P < 0.05, **P < 0.01, ***P < 0.001. (E, F) NB4 and NB4-R1 cells were incubated with 60 nM CBG, and immunofluorescence was performed to observe CHOP protein. Results are expressed as mean ± SD (n = 3).

**Figure 6.**
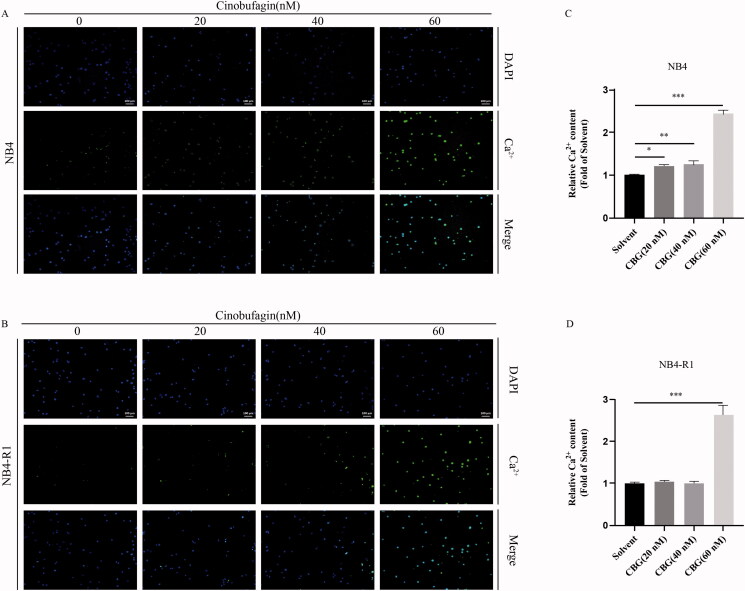
CBG induced Ca^2+^ overload in NB4 and NB4-R1 cells. NB4 and NB4-R1 cells were treated with different concentrations of CBG. (A, B) The intracellular Ca^2+^ level was detected by Fluo 4 probe and observed by fluorescence microscope. (C, D) Calcium was examined by using Fluo 4 and detected by the fluorescence quantitative reader. The results are exhibited as mean ± SD (n = 3). *P < 0.05, **P < 0.01, ***P < 0.001.

ER stress is often associated with apoptosis (Fernández et al. 2015). ER stress modulates three pathways: inositol-requiring enzyme 1 (IRE 1), PERK, and ATF6 (Oh et al. 2021). Western blotting revealed that CBG activated the PERK-eIF2α-ATF4 pathway (Figure 5(A,B)). Moreover, the expression levels of CHOP, an ER stress marker, were significantly increased (Figure 5(C–F)). Maintaining calcium homeostasis is one of the main functions of the ER, and ER stress often affects intracellular Ca^2+^ homeostasis (Kim and Kim 2018). Notably, we detected intracellular Ca^2+^ overload using a fluorescence microplate reader (Figure 6(A,B)) and fluorescence microscope (Figure 6(C,D)). These results suggested that CBG induced ER stress in NB4 and NB4-R1 cells.

### CBG-induced PML-RARA degradation was caspase-dependent

**Figure 7.**
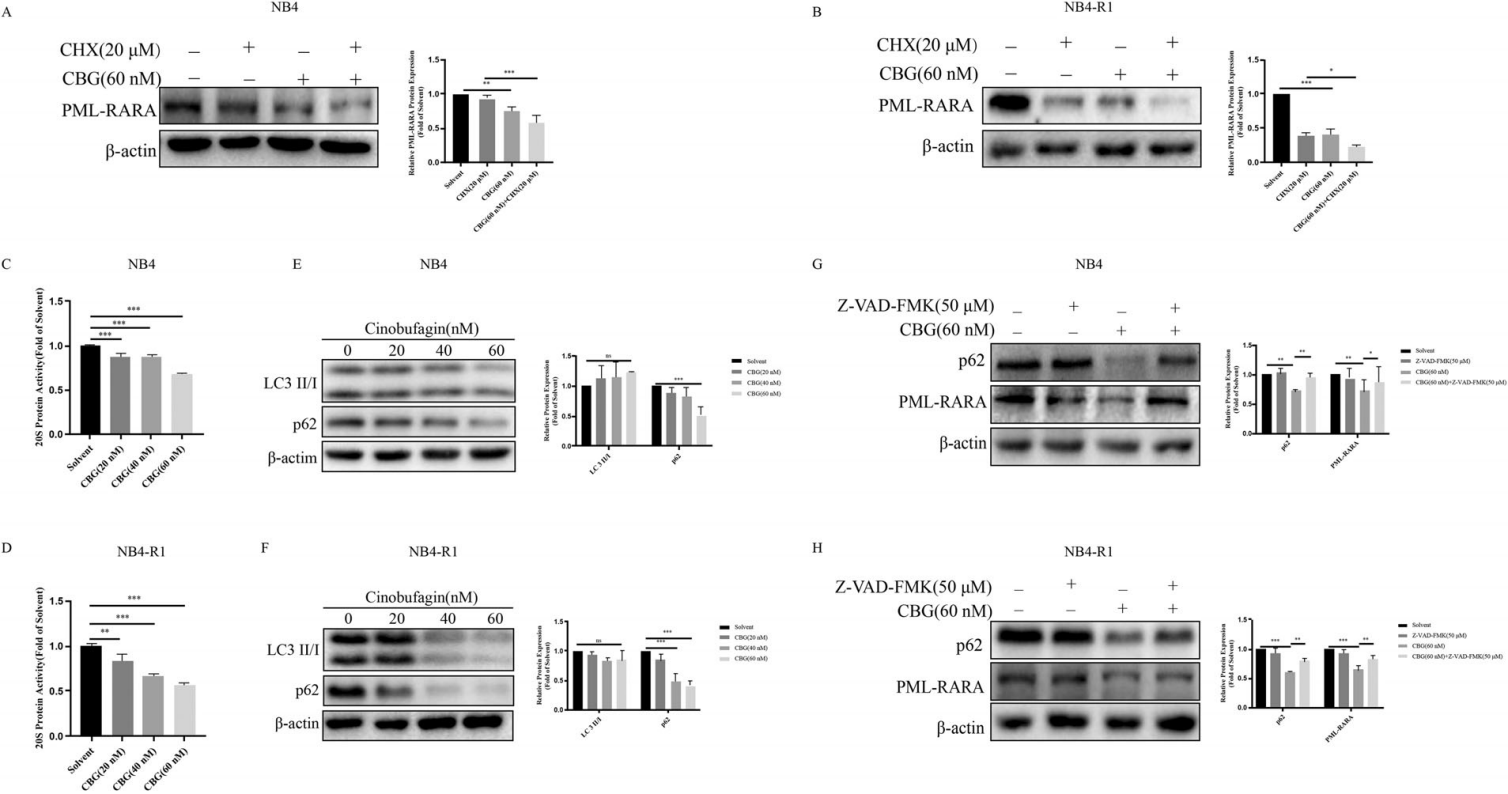
CBG-induced PML-RARA degradation in a caspase-dependent manner. (A, B) The cells were incubated with CHX (20 μM) for 2 h and then treated with 60 nM CBG for 24 h to detect the PML-RARA by western blotting. The results are expressed as mean ± SD (n = 3). Compared with the CBG group with a specified dose, *P < 0.05, **P < 0.01, ***P < 0.001. (C, D) Cells were incubated with CBG (0-60 nM) for 24 h, and then collected for 20S protease activity determination. (E, F) The expression levels of LC3 and p62 were detected by western blotting. Compared with solvent group, *P < 0.05, **P < 0.01, ***P < 0.001, ns: no significant difference. (G, H) Western blotting was used to detect the expression of p62 and PML-RARA after adding Z-VAD-FMK. Results are expressed as mean ± SD (n = 3). Compared with the CBG group with a specified dose, *P < 0.05, **P < 0.01, ***P < 0.001.

APL pathogenesis involves a chromosomal translocation, which generates the PML-RARA fusion protein (Yilmaz et al. 2021). Based on the above-mentioned experimental results, CBG effectively inhibited PML-RARA expression (Figure 2(A,B)). To explore the mechanism by which CBG inhibited the expression of PML-RARA fusion protein, we used CHX, a broad-spectrum protein synthesis inhibitor, in combination with CBG. Intriguingly, the combination of CHX and CBG inhibited PML-RARA expression significantly more strongly than CHX alone did (Figure 7(A,B)). PML-RARA is degraded via three pathways: the ubiquitin-proteasome (Rabellino et al. 2012), autophagy-lysosome (Isakson et al. 2010), and caspase-dependent (Wang et al. 2016) pathways. To further explore the mechanism by which CBG promoted PML-RARA fusion protein degradation, we conducted proteasome activity experiments (Figure 7(C,D)) and western blotting for autophagy-related proteins LC3 and p62 (Figure 7(E,F)). Notably, we found that LC3 II/I expression levels did not increase in NB4 and NB4-R1 cells after CBG treatment (Figure 7(E,F)). Additionally, p62 degradation may be related to caspase (Zhao et al. 2020). Therefore, we added Z-VAD-FMK and confirmed that p62 degradation was related to caspase (Figure 7(G,H)), which excluded autophagy, indicating that CBG promoted PML-RARA degradation independently of the ubiquitin-proteasome and lysosome pathways. Subsequently, we found that the CBG-induced degradation of PML-RARA fusion protein was significantly inhibited after the addition of Z-VAD-FMK (Figure 7(G,H)). These data demonstrated that CBG induced PML-RARA fusion protein in a caspase-dependent manner.

### CBG inhibited the β-catenin pathway

**Figure 8.**
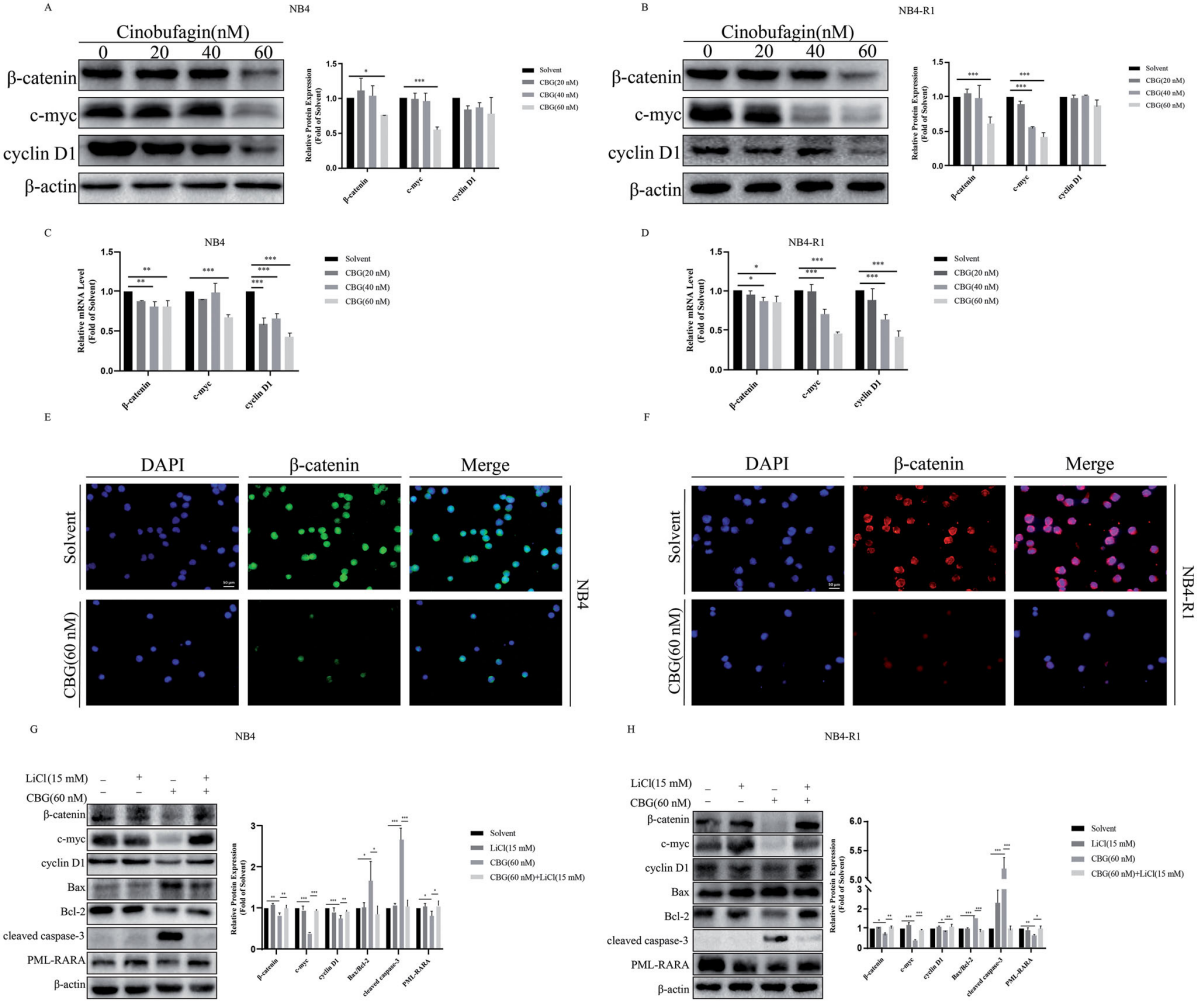
CBG inhibited the β-catenin signalling pathway. (A, B) The expression of β-catenin and its downstream target proteins c-myc and cyclin D1 were detected by western blotting. (C, D) The mRNA levels of *β-catenin*, *c-myc* and *cyclin D1* were detected by RT-PCR. Compared with solvent group, *P < 0.05, **P < 0.01, ***P < 0.001. (E, F) CBG treated NB4 and NB4-R1 cells for 24 h, the expression of β-catenin protein was determined by immunofluorescence assay. (G, H) After preincubation with LiCl, a β-catenin activator, for 2 h, CBG was treated with 24 h to detect the protein expression by western blotting. The results are exhibited as mean ± SD (n = 3). Compared with the CBG group with a specified dose, *P < 0.05, **P < 0.01, ***P < 0.001.

The β-catenin signalling pathway is extensively involved in various intracellular physiological functions such as cell invasion, migration (Li et al. 2020), differentiation (Zhao et al. 2020) and apoptosis (Zhang et al. 2018). Our data showed that CBG inhibited the expression of β-catenin and its downstream target protein c-myc and cyclin D1 (Figure 8(A–D)). Immunofluorescence assay also revealed that β-catenin expression levels significantly decreased after 24 h of treatment with 60 nM CBG (Figure 8(E,F)). We found that CBG alone increased the Bax/Bcl-2 ratio and cleaved caspase-3 expression levels and inhibited PML-RARA expression (Figure 2(A,B)). However, CBG in combination with LiCl, a β-catenin activator, reversed the increase in Bax/Bcl-2 ratio and cleaved caspase-3 expression levels and the CBG-inhibited PML-RARA expression (Figure 8(G,H)). Therefore, the β-catenin pathway was likely involved in CBG-induced apoptosis and PML-RARA degradation in NB4 and NB4-R1 cells.

## Discussion

APL is characterized by a chromosomal translocation which generates the PML-RARA fusion protein (Fasan et al. 2017). Early, untreated APL is a rapidly progressing condition with a high fatality rate. The combination of ATO and ATRA is often used as a clinical treatment for APL. However, adverse reactions, such as leukocytosis and infection, caused by ATO and ATRA may worsen the prognosis of patients with APL, and some patients are prone to relapse and ATRA resistance. Therefore, a highly efficient natural substance with minimal toxicity that can overcome ATRA resistance is required. CBG is an active substance in toad venom, a traditional Chinese medicine, that has been widely used to treat various cancers. However, only a few reports on CBG treatment of APL are available. This study demonstrated the *in vitro* effects and mechanisms of action of CBG on APL.

Previous studies have shown that CBG treats cancer by promoting apoptosis (Zhang L et al. 2020), autophagy (Ma et al. 2016) and cell cycle arrest (Pan et al. 2019). First, we found that CBG inhibited the viability of NB4 and NB4-R1 cells in a time- and dose-dependent manner. CBG is cardiotoxic (Xiong et al. 2019), therefore, we performed experiments with a myocardial cell line (H9C2 cells) and found no significant effects at CBG concentrations of 0-60 nM. Our data suggested that CBG inhibited the viability of NB4 and NB4-R1cells without cardiotoxic at these doses.

Mitochondrial damage is an important feature of apoptosis. Moreover, decreased MMP is a common sign of mitochondrial damage (Zhu et al. 2021). Mitochondria are the main sites of ROS, and massive ROS production may reflect mitochondrial damage. In the present study, CBG induced caspase-dependent apoptosis in NB4 and NB4-R1 cells. We experimentally confirmed that CBG induced ROS accumulation and mitochondrial damage.

Several natural products that induce ER stress and apoptosis, thereby exerting antitumor effects, have been discovered in recent years (Kim and Kim 2018). ER stress caused by intracellular misfolded and unfolded proteins can activate unfolded protein response via three pathways: the IRE 1, PERK, ATF6 (Pak et al. 2019). Simultaneously, ER stress is accompanied by an imbalance in cytoplasmic calcium homeostasis. Notably, we found that CBG activated the PERK-eIF2α-ATF4 pathway, causing an increase in intracellular Ca^2+^, which confirmed that CBG triggered ER stress in NB4 and NB4-R1 cells. ER stress-induced apoptosis is mainly mediated by CHOP which is activated by the PERK-eIF2α-ATF4 axis. In the present study, CHOP expression was increased. Particularly, CHOP regulates Bax and Bcl-2 expression (Alshammari et al. 2021; Chen et al. 2021; Hardy et al. 2021; Zheng et al. 2021), activates downstream caspase, and induces apoptosis (Hsu et al. 2018; Huang et al. 2018; Isobe et al. 2019). In the present study, we found that the Bax/Bcl-2 ratio increased following CBG administration.

APL is characterized by the presence of PML-RARA fusion protein (Yilmaz et al. 2021). In the present study, PML-RARA was inhibited. We used CHX, a broad-spectrum protein synthesis inhibitor, to inhibit PML-RARA synthesis and confirmed that the combination of CBG and CHX significantly enhanced the inhibition of PML-RARA expression compared to CHX alone, suggesting that CBG might promote PML-RARA degradation. Particularly, PML-RARA is degraded via the ubiquitin–proteasome (Zhu et al. 1999; Kopf et al. 2000; Nasr et al. 2009), autophagy–lysosomal pathway and caspase-dependent (Zhu et al. 1999; Nasr et al. 2009) pathways. Noteworthy, we found that CBG induced apoptosis and that caspase was a key target of CBG; therefore, we speculated that CBG-induced PML-RARA degradation might be related to caspase activation. To explore the pathway by which CBG induced the degradation of PML-RARA fusion protein, we performed a proteasome activity assay and found that CBG effectively inhibited proteasome activity. Additionally, we performed western blotting and detected the expression of autophagy-related proteins, LC3, and p62. Generally, LC3 II/I expression increased during autophagy, reflecting autophagosome formation (Kabeya et al. 2000), and the expression of p62, an autophagy degradation substrate (Islam et al. 2018), was decreased. We found that LC3 II/I expression was not increased, indicating the absence of autophagy. Interestingly, p62 expression was decreased; in particular, the cleavage of p62 is related to caspase activation and is involved in apoptosis (Zhao et al. 2020). To further explore PML-RARA degradation, we treated the cells with Z-VAD-FMK, a broad-spectrum caspase inhibitor, which inhibited PML-RARA degradation. These results suggested that CBG degraded the PML-RARA fusion protein in a caspase-dependent manner, but not via autophagy-lysosome and ubiquitin-proteasome pathways. Interestingly, the decrease in p62 expression was reversed after the addition of Z-VAD-FMK; therefore, we speculated that p62 degradation was autophagy-independent and possibly caspase-dependent.

The β-catenin signalling pathway is involved in various physiological activities, such as differentiation, invasion, migration, and apoptosis (Zhang et al. 2020). According to our results, CBG inhibited the expression of β-catenin and its downstream factors, c-myc and cyclin D1. Notably, LiCl, a β-catenin activator, reversed the CBG-induced increase in the Bax/Bcl-2 ratio and in cleaved caspase-3, as well as the CBG-induced decrease in PML-RARA expression. Nevertheless, determining whether β-catenin induced apoptosis by regulating ER stress or mitochondrial damage requires further investigation.

## Conclusion

**Figure 9.**
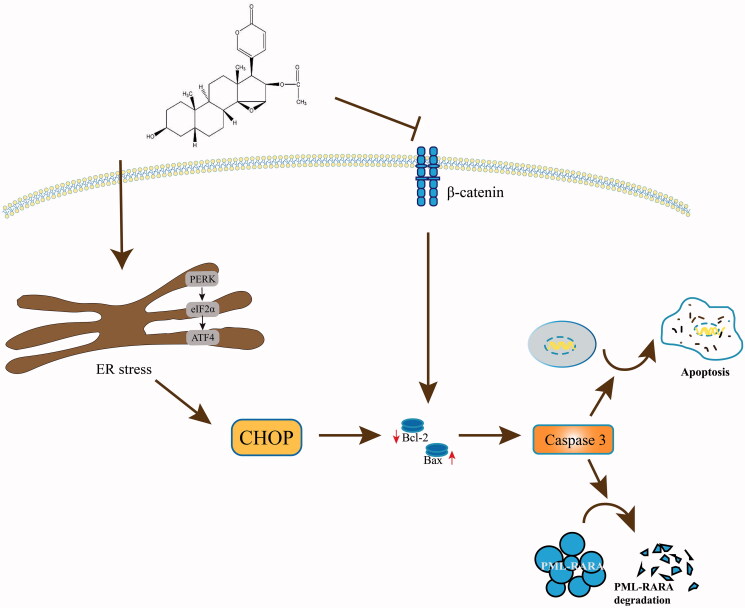
A schematic illustration of CBG-induced apoptosis and PML-RARA degradation in NB4 and NB4-R1 cells.

This study indicated that CBG induced NB4 and NB4-R1 cell apoptosis and PML-RARA degradation in a caspase-dependent manner, and that the β-catenin signalling pathway played a crucial role in this process (Figure 9). Therefore, CBG might be a potential candidate treatment for APL and ATRA-resistant APL.

## References

[CIT0001] Alshammari GM, Al-Qahtani WH, AlFaris NA, Albekairi NA, Alqahtani S, Eid R, Yagoub AEA, Al-Harbi LN, Yahya MA. 2021. Quercetin alleviates cadmium chloride-induced renal damage in rats by suppressing endoplasmic reticulum stress through SIRT1-dependent deacetylation of Xbp-1s and eIF2alpha. Biomed Pharmacother. 141:111862. 34246189 10.1016/j.biopha.2021.111862

[CIT0002] Chen L, Nie P, Yao L, Tang Y, Hong W, Liu W, Fu F, Xu H. 2021. TiO2 NPs induce the reproductive toxicity in mice with gestational diabetes mellitus through the effects on the endoplasmic reticulum stress signaling pathway. Ecotoxicol Environ Saf. 226:112814. 34592519 10.1016/j.ecoenv.2021.112814

[CIT0003] Fasan A, Haferlach C, Perglerovà K, Kern W, Haferlach T. 2017. Molecular landscape of acute promyelocytic leukemia at diagnosis and relapse. Haematologica. 102(6):e222–e224. 28341736 10.3324/haematol.2016.162206 PMC5451348

[CIT0004] Fernández A, Ordóñez R, Reiter RJ, González-Gallego J, Mauriz JL. 2015. Melatonin and endoplasmic reticulum stress: relation to autophagy and apoptosis. J Pineal Res. 59(3):292–307. 26201382 10.1111/jpi.12264

[CIT0005] Ge DZ, Sheng Y, Cai X. 2014. Combined staurosporine and retinoic acid induces differentiation in retinoic acid resistant acute promyelocytic leukemia cell lines. Sci Rep. 4:4821. 24769642 10.1038/srep04821 PMC4001092

[CIT0006] Hardy DB, Mu X, Marchiori KS, Mottola MF. 2021. Exercise in pregnancy increases placental angiogenin without changes in oxidative or endoplasmic reticulum stress. Med Sci Sports Exerc. 53(9):1846–1854. 33756523 10.1249/MSS.0000000000002647

[CIT0007] Hsu HY, Lin TY, Hu CH, Shu DTF, Lu MK. 2018. Fucoidan upregulates TLR4/CHOP-mediated caspase-3 and PARP activation to enhance cisplatin-induced cytotoxicity in human lung cancer cells. Cancer Lett. 432:112–120. 29746926 10.1016/j.canlet.2018.05.006

[CIT0008] Huang H, An Y, Jiao W, Wang J, Li S, Teng X. 2018. CHOP/caspase-3 signal pathway involves in mitigative effect of selenium on lead-induced apoptosis via endoplasmic reticulum pathway in chicken testes. Environ Sci Pollut Res Int. 25(19):18838–18845. 29713980 10.1007/s11356-018-1950-1

[CIT0009] Isakson P, Bjørås M, Bøe SO, Simonsen A. 2010. Autophagy contributes to therapy-induced degradation of the PML/RARA oncoprotein. Blood. 116(13):2324–2331. 20574048 10.1182/blood-2010-01-261040

[CIT0010] Islam MA, Sooro MA, Zhang P. 2018. Autophagic Regulation of p62 is Critical for Cancer Therapy. IJMS. 19(5):1405. 10.3390/ijms19051405 PMC5983640 29738493

[CIT0011] Isobe T, Tange S, Tasaki H, Kanamori K, Kato A, Nakanishi A. 2019. Upregulation of CHOP participates in caspase activation and virus release in human astrovirus-infected cells. J Gen Virol. 100(5):778–792. 30912739 10.1099/jgv.0.001250

[CIT0012] Kabeya Y, Mizushima N, Ueno T, Yamamoto A, Kirisako T, Noda T, Kominami E, Ohsumi Y, Yoshimori T. 2000. LC3, a mammalian homologue of yeast Apg8p, is localized in autophagosome membranes after processing. Embo J. 19(21):5720–5728. 11060023 10.1093/emboj/19.21.5720 PMC305793

[CIT0013] Kim C, Kim B. 2018. Anti-cancer natural products and their bioactive compounds inducing ER stress-mediated apoptosis: a review. Nutrients. 10(8):1021. 10.3390/nu10081021 PMC6115829 30081573

[CIT0014] Kim GH, Fang XQ, Lim WJ, Park J, Kang TB, Kim JH, Lim JH. 2020. Cinobufagin suppresses melanoma cell growth by inhibiting LEF1. IJMS. 21(18):6706. 10.3390/ijms21186706 PMC7554883 32933177

[CIT0015] Kogan SC, Bishop JM. 1999. Acute promyelocytic leukemia: from treatment to genetics and back. Oncogene. 18(38):5261–5267. 10498878 10.1038/sj.onc.1202996

[CIT0016] Kopf E, Plassat JL, Vivat V, de Thé H, Chambon P, Rochette-Egly C. 2000. Dimerization with retinoid X receptors and phosphorylation modulate the retinoic acid-induced degradation of retinoic acid receptors alpha and gamma through the ubiquitin-proteasome pathway. J Biol Chem. 275(43):33280–33288. 10869350 10.1074/jbc.M002840200

[CIT0017] Li L, Peng W, Zhou Q, Wan JP, Wang XT, Qi HB. 2020. LRP6 regulates Rab7-mediated autophagy through the Wnt/β-catenin pathway to modulate trophoblast cell migration and invasion. J Cell Biochem. 121(2):1599–1609. 31544984 10.1002/jcb.29394

[CIT0018] Li X, Chen C, Dai Y, Huang C, Han Q, Jing L, Ma Y, Xu Y, Liu Y, Zhao L, et al. 2019. Cinobufagin suppresses colorectal cancer angiogenesis by disrupting the endothelial mammalian target of rapamycin/hypoxia-inducible factor 1α axis. Cancer Sci. 110(5):1724–1734. 30839155 10.1111/cas.13988 PMC6501006

[CIT0019] Liang C, Ding M, Weng XQ, Sheng Y, Wu J, Li ZY, Cai X. 2019. Combination of enzastaurin and ATRA exerts dose-dependent dual effects on ATRA-resistant acute promyelocytic leukemia cells. Am J Cancer Res. 9(5):906–926. 31218101 PMC6556610

[CIT0020] Liu Y, Yu S, Xing X, Qiao J, Yin Y, Wang J, Liu M, Zhang W. 2021. Ginsenoside Rh2 stimulates the production of mitochondrial reactive oxygen species and induces apoptosis of cervical cancer cells by inhibiting mitochondrial electron transfer chain complex. Mol Med Rep. 24(6):873. 34713297 10.3892/mmr.2021.12513 PMC8569524

[CIT0021] Ma K, Zhang C, Huang MY, Li WY, Hu GQ. 2016. Cinobufagin induces autophagy-mediated cell death in human osteosarcoma U2OS cells through the ROS/JNK/p38 signaling pathway. Oncol Rep. 36(1):90–98. 27176794 10.3892/or.2016.4782 PMC4899018

[CIT0022] Nasr R, Lallemand-Breitenbach V, Zhu J, Guillemin MC, de Thé H. 2009. Therapy-induced PML/RARA proteolysis and acute promyelocytic leukemia cure. Clin Cancer Res. 15(20):6321–6326. 19808868 10.1158/1078-0432.CCR-09-0209

[CIT0023] Noguera NI, Catalano G, Banella C, Divona M, Faraoni I, Ottone T, Arcese W, Voso MT. 2019. Acute promyelocytic leukemia: update on the mechanisms of leukemogenesis, resistance and on innovative treatment strategies. Cancers (Basel). 11(10):1591. 10.3390/cancers11101591 PMC6826966 31635329

[CIT0024] Oh H, Kang MK, Park SH, Kim DY, Kim SI, Oh SY, Na W, Shim JH, Lim SS, Kang YH. 2021. Asaronic acid inhibits ER stress sensors and boosts functionality of ubiquitin-proteasomal degradation in 7β-hydroxycholesterol-loaded macrophages. Phytomedicine. 92:153763. 34601222 10.1016/j.phymed.2021.153763

[CIT0025] Pak S, Park S, Kim Y, Park JH, Park CH, Lee KJ, Kim CS, Ahn H. 2019. The small molecule WNT/β-catenin inhibitor CWP232291 blocks the growth of castration-resistant prostate cancer by activating the endoplasmic reticulum stress pathway. J Exp Clin Cancer Res. 38(1):342. 31387608 10.1186/s13046-019-1342-5 PMC6685284

[CIT0026] Pan Z, Luo Y, Xia Y, Zhang X, Qin Y, Liu W, Li M, Liu X, Zheng Q, Li D. 2020. Cinobufagin induces cell cycle arrest at the S phase and promotes apoptosis in nasopharyngeal carcinoma cells. Biomed Pharmacother. 122:109763. 31918288 10.1016/j.biopha.2019.109763

[CIT0027] Pan Z, Zhang X, Yu P, Chen X, Lu P, Li M, Liu X, Li Z, Wei F, Wang K, et al. 2019. Cinobufagin induces cell cycle arrest at the G2/M phase and promotes apoptosis in malignant melanoma cells. Front Oncol. 9:853. 31552178 10.3389/fonc.2019.00853 PMC6738445

[CIT0028] Rabellino A, Carter B, Konstantinidou G, Wu SY, Rimessi A, Byers LA, Heymach JV, Girard L, Chiang CM, Teruya-Feldstein J, et al. 2012. The SUMO E3-ligase PIAS1 regulates the tumor suppressor PML and its oncogenic counterpart PML-RARA. Cancer Res. 72(9):2275–2284. 22406621 10.1158/0008-5472.CAN-11-3159 PMC3342450

[CIT0029] Wang X, Lin Q, Lv F, Liu N, Xu Y, Liu M, Chen Y, Yi Z. 2016. LG-362B targets PML-RARα and blocks ATRA resistance of acute promyelocytic leukemia. Leukemia. 30(7):1465–1474. 27012866 10.1038/leu.2016.50

[CIT0030] Xiong X, Lu B, Tian Q, Zhang H, Wu M, Guo H, Zhang Q, Li X, Zhou T, Wang Y. 2019. Inhibition of autophagy enhances cinobufagin‑induced apoptosis in gastric cancer. Oncol Rep. 41(1):492–500. 30542704 10.3892/or.2018.6837

[CIT0031] Yamakawa H, Setoguchi S, Goto S, Watase D, Terada K, Nagata-Akaho N, Toki E, Koga M, Matsunaga K, Karube Y, et al. 2021. Growth inhibitory effects of ester derivatives of menahydroquinone-4, the reduced form of vitamin K(2(20)), on all-*trans* retinoic acid-resistant HL60 cell line. Pharmaceutics. 13(5):758. 34065416 10.3390/pharmaceutics13050758 PMC8161027

[CIT0032] Yilmaz M, Kantarjian H, Ravandi F. 2021. Acute promyelocytic leukemia current treatment algorithms. Blood Cancer J. 11(6):123. 34193815 10.1038/s41408-021-00514-3 PMC8245494

[CIT0033] Zeng CW, Chen ZH, Zhang XJ, Han BW, Lin KY, Li XJ, Wei PP, Zhang H, Li Y, Chen YQ. 2014. MIR125B1 represses the degradation of the PML-RARA oncoprotein by an autophagy-lysosomal pathway in acute promyelocytic leukemia. Autophagy. 10(10):1726–1737. 25126724 10.4161/auto.29592 PMC4198358

[CIT0034] Zhang L, Cheng H, Yue Y, Li S, Zhang D, He R. 2018. H19 knockdown suppresses proliferation and induces apoptosis by regulating miR-148b/WNT/β-catenin in ox-LDL -stimulated vascular smooth muscle cells. J Biomed Sci. 25(1):11. 29415742 10.1186/s12929-018-0418-4 PMC5804091

[CIT0035] Zhang L, Huang X, Guo T, Wang H, Fan H, Fang L. 2020. Study of cinobufagin as a promising anticancer agent in uveal melanoma through intrinsic apoptosis pathway. Front Oncol. 10:325. 32300551 10.3389/fonc.2020.00325 PMC7142239

[CIT0036] Zhang Z, Lv Z, Zhang W, Guo M, Li C. 2020. A novel β-catenin from *Apostichopus japonicus* mediates *Vibrio splendidus*-induced inflammatory-like response. Int J Biol Macromol. 156:730–739. 32311399 10.1016/j.ijbiomac.2020.04.103

[CIT0037] Zhao SJ, Kong FQ, Jie J, Li Q, Liu H, Xu AD, Yang YQ, Jiang B, Wang DD, Zhou ZQ, et al. 2020. Macrophage MSR1 promotes BMSC osteogenic differentiation and M2-like polarization by activating PI3K/AKT/GSK3β/β-catenin pathway. Theranostics. 10(1):17–35. 31903103 10.7150/thno.36930 PMC6929615

[CIT0038] Zhao Y, Zhu Q, Bu X, Zhou Y, Bai D, Guo Q, Gao Y, Lu N. 2020. Triggering apoptosis by oroxylin A through caspase-8 activation and p62/SQSTM1 proteolysis. Redox Biol. 29:101392. 31926620 10.1016/j.redox.2019.101392 PMC6909190

[CIT0039] Zheng Y, Zhang B, Guan H, Jiao X, Yang J, Cai J, Liu Q, Zhang Z. 2021. Selenium deficiency causes apoptosis through endoplasmic reticulum stress in swine small intestine. Biofactors. 47(5):788–800. 34128579 10.1002/biof.1762

[CIT0040] Zhu J, Gianni M, Kopf E, Honoré N, Chelbi-Alix M, Koken M, Quignon F, Rochette-Egly C, de Thé H. 1999. Retinoic acid induces proteasome-dependent degradation of retinoic acid receptor alpha (RARalpha) and oncogenic RARalpha fusion proteins. Proc Natl Acad Sci U S A. 96(26):14807–14812. 10611294 10.1073/pnas.96.26.14807 PMC24729

[CIT0041] Zhu L, Wang Y, Lv W, Wu X, Sheng H, He C, Hu J. 2021. Schizandrin A can inhibit non‑small cell lung cancer cell proliferation by inducing cell cycle arrest, apoptosis and autophagy. Int J Mol Med. 48(6):214. 34643254 10.3892/ijmm.2021.5047 PMC8522958

